# An examination of the social perceptions and vaping preferences of young electronic nicotine delivery system users

**DOI:** 10.3389/fpubh.2023.1150368

**Published:** 2023-03-30

**Authors:** Myles Davidson, Mohammed Al-Hamdani

**Affiliations:** ^1^Department of Psychology, Saint Mary's University, Halifax, NS, Canada; ^2^Department of Public Health, College of Health Science, QU Health, Qatar University, Doha, Qatar

**Keywords:** e-cigarette (e-cig), tobacco, young users, social aspects, vaping preferences

## Abstract

**Background:**

Little research has been conducted on social aspects and preferences of electronic nicotine delivery system (ENDS) use among young ENDS users, and none have examined differences in these aspects and preferences by gender and tobacco use status.

**Methods:**

A total of 558 young regular vapers (ages 16–24; vaped at least once a week for the last 3 months) from Nova Scotia were recruited to complete a demographic and vaping questionnaire. A 2 x 3 study design was used to compare participants on social aspects and vaping preferences based on gender (male or female) and tobacco use status (never, former, or current smoker). Chi-square tests were used to determine significant differences, and Bonferroni tests were used to assess over- and under-representation within significant variables.

**Results:**

Current tobacco-using male vapers had a higher frequency of experiencing pressure to vape from friends and current employment as compared to females. Former and never tobacco-using male vapers had a higher frequency of parental awareness of their vaping behavior than females. Former tobacco-using female vapers had a higher frequency of being influenced to vape by others they know on social media than males. Both never and former tobacco-using females reported a higher frequency of exposure to vaping content on social media than males. Never tobacco-using female vapers preferred vape pen devices relative to males.

**Conclusions:**

Important gender differences by tobacco use status exist and demonstrate differential patterns of social influence for ENDS use and their experiences within this demographic.

## 1. Introduction

Electronic Nicotine Delivery System (ENDS) use among youth and young adults represents an emerging public health concern (1–3). Much of the literature on this topic has focused on the factors that contribute to ENDS use (4–7), the preferences and perceptions of users (8–12), and the harms of ENDS use (13–16). Most studies, however, have not examined gender differences in perceptions and experiences with ENDS. Those that have found more exposure of youth males to advertising compared to females (17), greater expectancy effects for young adult males vs. females (18), and a higher proportion of males who are dual ENDS and cigarette users (19).

In an effort to better understand the relationship between ENDS and cigarettes, some studies have examined the patterns of use and experiences of ENDS users and ENDS and tobacco co-users (5, 20), as well as the risk factors for mono and dual use (21, 22). For instance, there is evidence that ENDS use is not consistently associated with quitting smoking (23, 24), and where ENDS-assisted smoking abstinence was noted, the benefit dissipated beyond 12 months (25). Further, 80% of ex-smokers who used ENDS for cessation report continuing to vape after 1 year of abstinence (26). On the contrary, ENDS use is associated with smoking initiation (27, 28) and may place ENDS-using adolescents at risk of becoming dual ENDS and cigarette users (17, 29). With respect to vaping-related perceptions, there are differences among ENDS users depending on their tobacco use status—for example, ENDS users who never used tobacco and those who are tobacco co-users prefer flavored vaping products more than ENDS users who are former tobacco users (30). As for risk factors, ENDS and cigarettes were found to share some, but not all risk factors (22, 31).

Although the vaping literature provides some knowledge on differences among vapers by gender or tobacco use status, there are two important gaps in this literature. First, the extent of the relationship between ENDS use, gender, and tobacco in the literature is confined to prevalence (32, 33). Some studies have examined the odds of exposure to and engagement of youth to vaping-related content in social media but did not assess gender differences (34). In all, there is a lack of literature on the differences in social aspects of vaping by gender or tobacco use status. Understanding the social dynamic of vaping among those with different tobacco use statuses in each gender is important to best appreciate the relationship between vaping and tobacco use by gender. Similarly, it is important to understand gender differences by tobacco use group to best target selective policies and interventions toward these groups.

Second, there is a dearth of studies on the social aspects of vaping in general. Some studies have found that peer approval is important for vapers but not smokers (35). However, aspects like sharing behavior, parental awareness, employment status, and the relative importance of social influences on vaping is important to understand how social circles influence individual vaping behavior. In addition, very little research has been conducted on how the specific characteristics of ENDS, such as the type of device and the concentration of nicotine used, influence vaping behavior, especially in relation to gender and tobacco use status.

The current study closes some gaps in the literature by examining how vapers of different genders with differing histories of tobacco use vary in social aspects. The goal is to contribute to the scarce vaping literature on differences in social aspects by gender and tobacco use status and appreciate their potential translation into selective policies.

## 2. Materials and methods

### 2.1. Sample

Participants for this study were recruited using paid Facebook and Instagram ads targeting the demographic of interest. Those who interacted with the ads were redirected to a Qualtrics survey (36). To be eligible to participate in the survey, participants had to be between the ages of 16–24 years, living in Nova Scotia and regular vapers (have vaped at a frequency of at least once a week for the last 3 months). A total of 558 participants completed the survey.

### 2.2. Survey

Participants who took part in the study were asked to complete a questionnaire containing a series of closed-ended demographic- and vaping-related questions. These questions were previously used in studies of a similar nature (30, 37). The questionnaire was used to inform demographic variables including age (“What is your age?”), gender (“What is your gender?”), tobacco use history (“Have you ever used tobacco [e.g., cigarettes, cigarillos/little cigars, chewing tobacco/chew, snuff, snus]?”), employment status (“Are you currently employed?”), nicotine content of vaping device (“Do you know how much nicotine is in the vape juice you use?”), and device type (“What type of vape do you usually use?”), as well as social variables including peer pressure to vape (“Have your friends ever tried to pressure you to vape?”), parental awareness (“Do your parents/guardians know that you vape?”), sharing behavior (“Have you ever offered to share your vape with someone else [even for one puff]?”), strongest influence to start vaping (“What most heavily influenced you to start vaping?”), and ever exposure to social media vaping content (“Do you see people posting about vaping on social media [e.g., Instagram/Facebook posts, Snapchat/Snapchat stories, tweets]?”). If participants were between the ages of 16–18 years, they answered the question about parental awareness as the age of majority in Nova Scotia is 19. Participants who provided complete responses were given a $10 gift card to Starbucks. Those who participated in the survey and chose to provide their email were entered into a prize draw for a chance to win one of five $100 gift cards, regardless of survey completion. Ethics approval for the study was provided by Saint Mary's University (REB# 19–105).

### 2.3. Analysis

Chi-square tests were used to assess differences by gender (male vs. female) and tobacco use status (never, former, or current users) across eight aspects of vaping: (1) pressure from peers to vape, (2) parental awareness, (3) sharing behavior, (4) strongest influence to start vaping, (5) ever exposure to social media vaping content, (6) employment status, (7) device type, and (8) nicotine concentration. Significant differences in each outcome level were assessed using Bonferroni correction tests to determine over- and under-representation within each group. To conserve statistical power, differences were not assessed for any variables whose total sample size was <5. All statistical tests were completed using SPSS Version 25.

## 3. Results

The average age of the sample was 18.5 years (SD = 2.2). Table 1 presents the sample and vaping characteristics split by gender. Males comprised more of the sample (53.6%) than females (46.4%). In terms of tobacco use status, most of the sample (57%) were former tobacco users, whereas 28% were never tobacco users and 15% were current tobacco users. Most of the sample (76.9%) were employed at the time of the survey. In terms of vaping characteristics, the overwhelming majority (79.6%) of the sample reported using the highest available concentrations of nicotine when they vape (50–60 mg/mL). Similarly, most respondents (64.9%) reported using a pod-based device, whereas fewer reported using mod-based devices (22.4%), vape pens (11.8%), and traditional e-cigarettes (0.9%).

**Table 1:** Demographic and vaping characteristics of the sample.

**Variable**	**Total male, *N* (%)**	**Total female, *N* (%)**
**Tobacco use history**		
Never user	54 (18.1)	102 (39.4)
Former user	203 (67.9)	115 (44.4)
Current user	42 (14.0)	42 (16.2)
**Employment status**		
Currently employed	242 (80.9)	187 (72.2)
Unemployed	57 (19.1)	72 (27.8)
**Peer pressure to vape**		
No	207 (69.2)	167 (64.5)
Yes	92 (30.8)	92 (35.5)
**Parental knowledge of vaping**		
No	37 (33.9)	87 (58.0)
Yes	72 (66.1)	63 (42.0)
**Offered to share your vape**		
No	19 (6.4)	10 (3.9)
Yes	261 (87.2)	216 (83.4)
I do not own a vape	19 (6.4)	33 (12.7)
**Strongest influence to start vaping**		
Friends	177 (59.2)	178 (68.7)
Smoking cessation	74 (24.7)	42 (16.2)
Social media exposure	14 (4.7)	26 (10.0)
Family	9 (3.0)	3 (1.2)
Celebrity influencers	3 (1.0)	1 (0.4)
Advertisements	2 (0.7)	1 (0.4)
Other	20 (6.7)	8 (3.1)
**Social media exposure to vaping**		
No	83 (27.9)	32 (12.4)
Yes	214 (72.1)	226 (87.6)
**Nicotine concentration in vaping device**		
10–20 mg/mL	9 (3.3)	8 (3.9)
35 mg/mL	10 (3.7)	6 (2.9)
50–60 mg/mL	254 (93.0)	190 (93.2)
**Device type**		
e-cigarette	4 (1.3)	1 (0.4)
Vape pen	19 (6.4)	47 (18.2)
Mod	69 (23.1)	56 (21.6)
Pod	207 (69.2)	155 (59.8)

*N* = 558.

Table 2 explores the differences in social aspects of vaping, organized by gender and tobacco use status. There were gender differences in experiencing pressure to vape from peers among ENDS users that were current tobacco users [χ^2^ (df = 1, *p* = 0.018) = 5.57], but not those who were former tobacco [χ^2^ (df = 1, *p* = 0.059) = 3.57] or never tobacco [χ^2^ (df = 1, *p* = 0.864) = 0.03] users. Among ENDS users who currently use tobacco, a higher proportion of males reported peers pressuring them to vape relative to females.

**Table 2:** ENDS user differences in social aspects organized by gender and tobacco use history.

	**Never tobacco user**		**Former tobacco user**		**Current tobacco user**			
**Variable**	**Male**, ***n*** **(%)**	**Female**, ***n*** **(%)**	**Male**, ***n*** **(%)**	**Female**, ***n*** **(%)**	**Male**, ***n*** **(%)**	**Female**, ***n*** **(%)**	**Total male**, ***N*** **(%)**	**Total female**, ***N*** **(%)**
**Peer pressure to vape** ^*^								
No	32 (59.3)	59 (57.8)	151 (74.4)	74 (64.3)	24 (57.1)	34 (81.0)	207 (69.2)	167 (64.5)
Yes	22 (40.7)^i^	43 (42.2)^i^	52 (25.6)^i^	41 (35.7)^i^	18 (42.9)^i^	8 (19.0)^ii^	92 (30.8)	92 (35.5)
**Parental knowledge of vaping** ^*^								
No	15 (51.7)	53 (76.8)	18 (28.1)	27 (47.4)	4 (25.0)	7 (29.2)	37 (33.9)	87 (58.0)
Yes	14 (48.3)^i^	16 (23.2)^ii^	46 (71.9)^i^	30 (52.6)^ii^	12 (75.0)^i^	17 (70.8)^i^	72 (66.1)	63 (42.0)
**Offered to share your vape**								
No	4	2	11	5	4	3	19	10
Yes	44	76	183	105	34	35	261	216
I do not own a vape	6	24	9	5	4	4	19	33
**Strongest influence to start vaping** ^*^								
Friends	40 (74.1)^i^	83 (81.4)^i^	114 (86.2)^i^	75 (65.2)^i^	23 (54.8)^i^	20 (47.6)^i^	177 (59.2)	178 (68.7)
Smoking cessation	0 (0.0)^i^	1 (1.0)^i^	59 (29.1)^i^	25 (21.7)^i^	15 (35.7)^i^	16 (38.1)^i^	74 (24.7)	42 (16.2)
Social media exposure	8 (14.8)^i^	11 (10.8)^i^	6 (3.0)^i^	13 (11.3)^ii^	0 (0.0)^i^	2 (4.8)^i^	14 (4.7)	26 (10.0)
Family	2 (3.7)^i^	1 (1.0)^i^	7 (3.4)^i^	2 (1.7)^i^	0 (0.0)^i^	0 (0.0)^i^	9 (3.0)	3 (1.2)
Celebrity influencers	1 (1.9)	1 (1.0)	1 (0.5)	0 (0.0)	1 (2.4)	0 (0.0)	3 (1.0)	1 (0.4)
Advertisements	1 (1.9)	0 (0.0)	0 (0.0)	0 (0.0)	1 (2.4)	1 (2.4)	2 (0.7)	1 (0.4)
Other	2 (3.7)^i^	5 (4.9)^i^	16 (7.9)^i^	0 (0.0)^ii^	2 (4.8)^i^	3 (7.1)^i^	20 (6.7)	8 (3.1)
**Social media exposure to vaping** ^*^								
No	14 (25.9)	13 (12.7)	60 (29.9)	13 (11.4)	9 (21.4)	6 (14.3)	83 (27.9)	32 (12.4)
Yes	40 (74.1)^i^	89 (87.3)^ii^	141 (70.1)^i^	101 (88.6)^ii^	33 (78.6)^i^	36 (85.7)^i^	214 (72.1)	226 (87.6)
**Employment status** ^*^								
No	11 (20.4)	26 (25.5)	39 (19.2)	26 (22.6)	7 (16.7)	20(47.6)	57 (19.1)	72 (27.8)
Yes	43 (79.6)^i^	76 (74.5)^i^	164 (80.8)^i^	89 (77.4)^i^	35 (83.3)^i^	22 (52.4)^ii^	242 (80.9)	187 (72.2)
**Nicotine concentration**								
Low	0 (0.0)^i^	2 (2.9)^i^	9(4.7)^i^	5 (5.1)^i^	0 (0.0)^i^	1 (2.7)^i^	9 (3.3)	8 (3.9)
Medium	2 (4.4)^i^	3 (4.4)^i^	6 (3.1)^i^	3 (3.0)^i^	2 (5.4)^i^	0 (0.0)^i^	10 (3.7)	6(2.9)
High	43 (95.6)^i^	63 (92.6)^i^	176(92.1)^i^	91 (91.9)^i^	35(94.6)^i^	36 (97.3)^i^	254 (93.0)	190 (93.2)
**Device type** ^*^								
e-cigarette	2 (3.7)^i^	0(0.0)^i^	0 (0.0)^i^	1(0.9)^i^	2 (4.8)^i^	0 (0.0)^i^	4 (1.3)	1 (0.4)
Vape pen	2 (3.7)^i^	26 (25.5)^ii^	12(5.9)^i^	15 (13.0)^ii^	5 (11.9)^i^	6(14.3)^i^	19 (6.4)	47 (18.2)
Mod	13 (24.1)^i^	22 (21.6)^i^	49 (24.1)^i^	22 (19.1)^i^	7 (16.7)^i^	12(28.6)^i^	69 (23.1)	56 (21.6)
Pod	37 (68.5)^i^	54(52.9)^i^	142 (70.0)^i^	77 (67.0)^i^	28 (66.7)^i^	24 (57.1)^i^	207 (69.2)	155 (59.8)

Column percentages are included for variables that significantly varied by gender group. Each roman superscript (i and ii) denotes a subset of the variable that significantly differs by gender group. Categories with different roman superscripts denote column proportions that differ from each other at *p* < 0.05.

* Significant group differences at *p* < 0.05. *p*-values were Bonferroni corrected. *N* = 558.

In terms of parental awareness of vaping behavior, there were gender differences among ENDS users who never used tobacco [χ^2^ (df = 1, *p* = 0.014) = 6.05] and those who formerly used tobacco [χ^2^ (df = 1, *p* = 0.029) = 4.78], but not those who currently use tobacco [χ^2^ (df = 1, *p* = 0.772) = 0.084]. In both ENDS users who are never tobacco users and those who are former tobacco users, males reported a higher frequency of parental awareness of their vaping behavior relative to females.

There were no gender differences reported in sharing of vaping devices in the never [χ^2^ (df = 2, *p* = 0.056) = 5.78], former [χ^2^ (df = 2, *p* = 0.914) = 0.18], or current [χ^2^ (df = 2, *p* = 0.924) = 0.16] tobacco use groups.

In terms of the strongest influence to start vaping, there were gender differences among ENDS users who were former tobacco users [χ^2^ (df = 5, *p* < 0.001) = 21.46], but not never [χ^2^ (df = 6, *p* = 0.568) = 4.81] or current [χ^2^ (df = 5, *p* = 0.632) = 3.44] tobacco users. In ENDS users who are former tobacco users, females reported a higher frequency of influence by the vaping behavior of others they know on social media relative to males.

Gender differences were observed in exposure to vaping on social media among ENDS users who were never tobacco users [χ^2^ (df = 1, *p* = 0.038) = 4.29] and those who formerly used tobacco [χ^2^ (df = 1, *p* < 0.001) = 13.90], but not current tobacco users [χ^2^ (df = 1, *p* = 0.393) = 0.73]. For both never and former tobacco users, females reported a higher proportion of exposure to vaping content on social media relative to males.

In addition, gender differences were observed with respect to employment status for current tobacco users [χ^2^ (df = 1, *p* = 0.002) = 0.922], but not for never tobacco users [χ^2^ (df = 1, *p* = 0.47) =.51] or former tobacco users [χ^2^ (df = 1, *p* = 0.47) = 0.52]. Among ENDS users who currently co-use tobacco, male dual users were overrepresented in the employed group in comparison to their female counterparts.

There were no gender differences reported with respect to nicotine concentration in the never [χ^2^ (df = 2, *p* = 0.51) = 1.35], former [χ^2^ (df = 2, *p* = 0.99) = 0.02], or current [χ^2^ (df = 2, *p* = 0.22) = 3.01] tobacco use groups.

Lastly, gender differences were observed with respect to device type in the never tobacco user group [χ^2^ (df = 3, *p* = 0.002) = 14.68], but not in the former [χ^2^ (df = 3, *p* = 0.07) = 7.08] or current [χ^2^ (df = 3, *p* = 0.29) = 3.71] tobacco user groups. Among ENDS users with no prior use of tobacco, Females were overrepresented as vape pen users relative to their male counterparts.

## 4. Discussion

For all groups overall, peer pressure and parental awareness of vaping behavior are high. Friends are the strongest influence to start vaping, and smoking cessation is much less important for all groups except current ENDS and tobacco co-users. Finally, exposure to social media content concerning vaping is very high. These general trends are in line with past literature that demonstrates the importance of peer pressure in vaping initiation (38), the importance of smoking cessation for dual ENDS and cigarette users (5), and the high exposure of young vapers to vaping content on social media (39). Our findings are novel in that they differ from past literature showing smoking cessation as being the strongest influence to vape among adult dual users of tobacco and vaping (5), since the current study was conducted with youth and young adults, and the importance of smoking cessation as a reason to start vaping was evident in vapers who reported former (not current) tobacco use. Further, the current study suggests that friends are more frequently reported as a reason to start vaping in comparison to smoking cessation.

Vaper gender differences in social aspects are complex and highly dependent on their tobacco use status. Interestingly, these gender differences are observed among ENDS users of different tobacco use statuses depending on the social aspect in question. In general, male vapers appear to report being influenced by real social factors (peer pressure and parental awareness), whereas female vapers appear to be report being strongly influenced by virtual social factors (vaping behavior of friends on social media, high exposure to vaping content on social media). These categorical differences add a novel finding to the current literature and suggest that efforts to intervene with vaping behavior require an understanding of the differential cues for each gender. Increasing awareness of social media effects for young female vapers is key [e.g., (40)], while skill acquisition and parental education of vaping may serve male vapers well [e.g., (31)]. More research is needed to determine whether these differences in social aspects are predictive of ENDS use frequency and initiation. Specific gender differences in social aspects for each tobacco use subgroup will be discussed below in the context of the broader literature.

One of the gender differences observed among current ENDS and tobacco co-users is in peer pressure to vape where more males report friends pressuring them to vape relative to females. Perhaps this is related to the fact that male smokers smoke more cigarettes relative to females (41), and peer pressure is thus more likely in the form of encouraging males to adopt vaping behavior, a less harmful alternative. This logic is supported by the high percentage of current ENDS and tobacco users that report friends and smoking cessation as important factors for vaping initiation. The explanation is further supported by past literature that reveals the importance of smoking cessation to current ENDS and tobacco co-users specifically (5).

When it comes to parental awareness, male vapers report higher frequencies than female vapers for groups that never used tobacco or formerly used it. This result is consistent with the tendency of males to be more risk-taking and not as careful as females when it comes to discreetness [e.g., 31]. The potential reason for why no gender differences were observed among ENDS and tobacco co-users stems from the fact parents are likely to be aware of their tobacco use too given that tobacco emits a smell that is hard to hide. Further research is needed to determine the specific reason for this difference in parental awareness.

More female vapers perceive social media exposure to be the strongest influence for vaping initiation in comparison to male vapers. Within the former tobacco user subgroup, female vapers report exposure to social media as the strongest influence for vaping compared to male vapers. This finding underscores the importance of social media in influencing the vaping behavior of female vapers. This is in line with past research that shows the importance of social media content, like tricks for females specifically [e.g., 30]. This study adds the novel finding that the importance of this content depends on tobacco use status. It is not surprising to not see a gender difference in social media factors among current ENDS and tobacco users given the importance of other aspects to them [e.g., friends, smoking cessation; (17)]. Considering that previous literature has found a link between social media exposure to vaping and vaping use and expectancies (42), this study highlights the need to consider both gender and tobacco use status when examining the relationships between social media and ENDS use to determine whether use patterns and expectancies differ based on these factors.

With respect to employment status, this study found that more male dual users of tobacco and ENDS were employed compared to female dual users. One possible mechanism that may explain the difference in employment between male and female dual tobacco and e-cigarette users is that males may rely on both ENDS and tobacco use to deal with work pressures. Similar findings have been found in the literature with respect to coping, especially in the context of stress-inducing events (43). Future research should seek to determine whether this finding is stable across different samples.

In terms of devices, females who use ENDS only were shown to prefer vape pens relative to males. This is in sync with the tobacco literature, where females tend to use slim cigarettes more than males, as vape pens are slim and feminine-laden compared to other devices (44). It is pertinent to explore this finding in more detail to determine whether the design of the vape pen is the driving force behind this discrepancy, or whether some other aspect of the device makes it desirable for female vapers.

Lastly, despite a lack of statistically significant findings, it should be noted that over three-quarters of the sample reported using the highest available concentrations of nicotine in their vape juice. This finding is alarming given what is known about the mechanisms behind nicotine addiction, as well as the fact that the intensity of the addiction is a commonly cited barrier to quitting (45, 46). It is imperative that available nicotine concentrations are targeted by policymakers to try and curb vaping addiction in young people.

### 4.1. Limitations

There are number of limitations to this study. First, the results are not generalizable to other geographical regions because they are based on a sample from Nova Scotia. However, youth and young adults tend to share similar perceptions regardless of geographical region (35). Nevertheless, future studies in other geographical areas may be needed. Second, the study has a cross sectional design and did not examine changes in social aspects over time. Longitudinal studies are needed to better capture differences in social aspects toward vaping which may evolve as ENDS continue to evolve in their design and content. Third, the study was not an exhaustive examination of social aspects as it did not explore all aspects of sharing behavior, support from others to quit vaping, advertisements for vaping on social media, and others. Future studies should examine more untapped social aspects for a better understanding of how gender and tobacco status influence social aspects of vaping among ENDS users. Fourth, the current study was unable to examine differences in social aspects and preferences across ethnicities and diverse genders. Future studies should incorporate this diversity into their design. Fifth, there is a chance that some of the findings are due to the small sample size in some response categories, as well as the small number of individuals who reported being current tobacco users relative to never or former tobacco users. As such, these findings should be interpreted in light of some caution. Finally, the fact that the sample was collected using social media platforms could explain why the exposure to social media ads was high among ENDs users in this study. Caution should be exercised when interpreting the results pertaining to social media ads in this study as they may overestimate the exposure of ENDS users to ads. Future studies should compare social media ad exposure in samples collected from social media platforms vs. other methods to ascertain true levels of exposure to ads in the general population of ENDS users.

## 5. Conclusion

This investigation examined differences in social aspects of vaping among youth and young adult vapers based on gender and tobacco use status. The results demonstrate that meaningful gender differences can be discerned based on tobacco use status. In general, male current tobacco users appear to be influenced more by their peer group and employment, whereas female never and former tobacco users are more influenced by social media exposure to vaping content and device type. In all, these results suggest that various social aspects that may influence vaping behavior vary across genders and tobacco use status. More work is needed to assess how these differences, especially those related to parental knowledge, exposure to vaping on social media, employment status, and device type utilized, may explain differences in use behaviors like frequency of use, use for coping with stress, or initiation of use between male and female ENDS users. This largely unregulated landscape is ripe with opportunities for harm reduction and vaping prevention within this demographic.

## Data availability statement

The datasets presented in this article are not readily available because the ethics approval for the study limits use of the data for the specified objectives of the project which included this study. Requests to access the datasets should be directed to MA-H, mohammed.al-hamdani@smu.ca.

## Ethics statement

The studies involving human participants were reviewed and approved by Institutional Review Board, Saint Mary's University. Written informed consent from the participants' legal guardian/next of kin was not required to participate in this study in accordance with the national legislation and the institutional requirements.

## Author contributions

MA-H administered the project, supervised it, and was the awardee of the grant from the Nova Scotia Department of Health and Wellness. All authors contributed to the write up, analysis, and response to the comments from reviewers.
